# Risk factors for homicide victimization in post-genocide Rwanda: a population -based case- control study

**DOI:** 10.1186/s12889-015-2145-z

**Published:** 2015-08-21

**Authors:** Wilson Rubanzana, Joseph Ntaganira, Michael D. Freeman, Bethany L. Hedt-Gauthier

**Affiliations:** Department of Epidemiology and Biostatistics, University of Rwanda College of Medicine and Health Sciences, School of Public Health, Kigali, Rwanda; Rwanda National Police Directorate of Medical Service, Kigali, Rwanda; Department of Global Health and Social Medicine, Harvard Medical School, Boston, USA; Oregon Health & Science University, School of Medicine, Portland, Oregon USA; Umeå University, Faculty of Medicine, Section of Forensic Medicine, Umeå, Sweden; Department of Forensic Medicine, Aarhus University, Faculty of Health Sciences, Aarhus, Denmark

## Abstract

**Background:**

Homicide is one of the leading causes of mortality in the World. Homicide risk factors vary significantly between countries and regions. In Rwanda, data on homicide victimization is unreliable because no standardized surveillance system exists. This study was undertaken to identify the risk factors for homicide victimization in Rwanda with particular attention on the latent effects of the 1994 genocide.

**Methods:**

A population-based matched case–control study was conducted, with subjects enrolled prospectively from May 2011 to May 2013. Cases of homicide victimization were identified via police reports, and crime details were provided by law enforcement agencies. Three controls were matched to each case by sex, 5-year age group and village of residence. Socioeconomic and personal background data, including genocide exposure, were provided via interview of a family member or through village administrators. Conditional logistic regression, stratified by gender status, was used to identify risk factors for homicide victimization.

**Results:**

During the study period, 156 homicide victims were enrolled, of which 57 % were male and 43 % were female. The most common mechanisms of death were wounds inflicted by sharp instruments (knives or machetes; 41 %) followed by blunt force injuries (36.5 %). Final models indicated that risk of homicide victimhood increased with victim alcohol drinking patterns. There was a dose response noted for alcohol use: for minimal drinking versus none, adjusted odds ratio (aOR) = 3.1, 95%CI: 1,3–7.9; for moderate drinking versus none, aOR = 10.1, 95%CI: 3.7–24.9; and for heavy drinking versus none, aOR = 11.5, 95%CI: 3.6–36.8. Additionally, having no surviving parent (aOR = 2.7, 95%CI: 1.1–6.1), previous physical and/or sexual abuse (aOR = 28.1, 95%CI: 5.1–28.3) and drinking illicit brew and/or drug use (aOR = 7.7, 95%CI: 2.4–18.6) were associated with a higher risk of being killed. The test of interaction revealed that the variables that were significantly associated with a higher risk of homicide victimhood, did not exhibit any difference according to sex of the victim. However, the effect of belonging to a religion differed between women and men, but was significantly protective for both (aOR = 0.002, 95%CI: 0.001–0.054 and aOR = 0.20, 95%CI: 0.052–0.509, respectively).

**Conclusion:**

Homicide victims in Rwanda are relatively young and the proportion of female victims is one of the highest globally. Contrary to the initial study considerations, genocide exposure (either as a survivor or perpetrator) was not a significant predictor of homicide victimization. Rather, risk factors were similar to those described in other countries, regardless of gender status. Sensitizing communities against alcohol heavy drinking, and illicit brew drinking and/or drug abuse and physical or sexual violence could reduce the homicide rate in Rwanda.

## Background

Homicide is a global public health issue [1–5]. A variety of individual and societal factors have been identified as risk factors for homicide, including religion, education, participation in specific types of illegal activity, political instability, lack of arms regulation and the presence of endemic violence in the form of wars, ethnic cleansing and post-conflict effects [2–7]. The type and degree of homicide victimization risk greatly depends on culture and context [1–8]. To date, very few studies in sub-Saharan Africa have explored risk factors for homicide victimization.

The risk for homicide victimization in Rwanda is of particular interest because of the recent history of genocide. The 1994 genocide against ethnic Tutsi in Rwanda is one of the most notorious crimes against humanity in the second half of the twentieth century [9]. Over a period of approximately one hundred days as many as 1,000,000 Rwandans became victims of mass murder [10]. In post-genocide Rwanda, the National Police, which was established in 2000, started documenting counts of homicide in 2008, when 300 cases were recorded. In the following years, the number of cases varied, with 278 cases in 2009, 240 cases in 2010, 255 in 2011 and 373 cases in 2012 [11]. A number of homicides have been anecdotally linked to persisting genocide effects. Isolated cases in which Tutsi survivors were specifically targeted were reported across the country, prompting concern for the safety of surviving genocide victims. Concerns have also been raised in Rwanda that witnesses who provided testimony against charged perpetrators may be targets of homicide.

The only source of information in Rwanda on homicide victimization currently is the National Police, and these data are likely underreported due to insufficient resources for forensic investigation. For instance, in 2012, police reported an annual homicide rate of 3.5 per 100,000 [11]. This rate is considerably lower than the Rwanda estimate for the same year by the United Nations Office on Drugs and Crimes, which was 17.1 per 100,000 [12]. In addition to being uncertain of the accuracy of homicide rates reported by the National Police or the UN, neither group includes information regarding the role of genocide in the deaths.

In the present study we describe the characteristics of homicide victims in Rwanda and identify risk factors associated with being a victim of homicide in Rwanda. The purpose of this investigation is to serve as a first step in surveying the current state of homicide in Rwanda in order to develop strategies to reduce violent death and improve the health and wellbeing of the Rwanda population.

## Methods

A collaboration between police homicide investigators and public health researchers was used to conduct an epidemiological study on violent fatal injuries. In this study, we applied a matched case–control study design in a field-based setting to investigate risk factors associated with homicide victimization.

### Study subjects

In this study, victim of homicide refers to an adult person killed as a result of a murder or manslaughter as determined by the national police criminal investigation department. The Rwandan penal code defines murder as “the intentional killing of a person” that “shall be punishable by life imprisonment,” and manslaughter as “intentional killing even if the offender was mistaken about the person who was the victim or his/her plan would be fulfilled depending on circumstances beyond his/her control” [13]. Cases were prospectively identified homicide victims, 18 years or older and of Rwandan citizenship, who died between May 2011- May 2013 as reported by the Rwanda police criminal investigation department. Homicide victims were identified via daily reports of unnatural deaths from the National Police. Exclusion criteria included unconfirmed/unclear case of homicide, unidentified victim and inability to trace family members of the deceased.

Control subjects were Rwandan citizens selected from live residents 18 years of age and older. Controls were matched to cases by age (±5 years), gender and area of residence. For area of residence matching criteria, control subjects were drawn from residents of a village neighboring the village of the case in order to minimize information bias and avoid interference with an active police investigation. Each case was matched to three living controls, who were identified with the assistance of local village administrators who were blinded to the aim of the study.

### Data collection

Information on cases and control subjects was obtained from three sources: the investigating police officer, the study subjects’ family members and the village administrator. Information was collected within one week of the date of the homicide for both cases and controls. Homicide victim investigation began with the interviewer introducing himself to the investigating police officer and requesting his collaboration in the data collection. The victim identity and manner of death were ascertained and information on the circumstances leading to homicide was collected. This information included the location, time of the incident, killer-victim relationship, alleged motive of homicide, the cause of death, any medical assistance received by the victim prior to the death and whether a post-mortem examination was performed.

Afterwards, a home-based interview was conducted. A detailed piloted questionnaire was administered to the next of kin (first-degree relatives, where possible) of both cases and controls. It was designed in English and then translated into Kinyarwanda. The questionnaire collected data on socio-demographic characteristics, employment status, previously experienced gender based violence (that included physical assault, psychological harassment and/or sexual assault), alcohol drinking behavior, drug use, past criminal record and information regarding genocide exposure. With regard to genocide exposure, five characteristics were of interest: 1) being a survivor, defined as a Rwandan who was living in Rwanda during the genocide period (6 April-4 July 1994) who was targeted for violence because of his or her Tutsi ethnicity [14]; 2) having lost a first-degree family member to genocide; 3) having been convicted of genocide in a criminal or Gacaca traditional court [15]; 4) having a first-degree relative who was convicted of genocide in a criminal or Gacaca traditional court; and 5) being a witnes*s*, defined as testifying at a case of genocide in criminal court or Gacaca traditional courts. Classification into a genocide exposure category was based on responses by the victim’s next of kin or family relative.

Eligible study respondents were approached by investigators, who explained the purpose of the research, responded to questions and then asked for their voluntary participation in the study. The investigator administered the questionnaire verbally in a face-to-face setting. When the next of kin appeared to be unsure of the answer, further information was obtained from the lead police investigator or the village leader who keeps administrative records. This occurred specifically in some cases where illiterate next of kin did not know some demographic characteristics of the study subjects or intentionally did not give true information on the genocide involvement of their family members.

The information was collected by trained interviewers and recorded on a standard questionnaire form. The questions were tightly structured and closed-ended. Because of the nature and sensitivity of the study, the interviewers were police nurses who had skills in criminal investigations and counseling and great care was taken in the collection of these data. They first conveyed sympathy to the affected family, provided moral support and created a caring environment that allowed the next of kin or a close family member to willingly supply the needed information. All cases and controls relatives who were asked to participate in our study provided information.

### Statistical analysis

Data obtained from police reports were used to describe the characteristics of homicide victimization in Rwanda. Interview data collected on cases and controls were used for risk assessment in a multivariable conditional logistic regression model. The first step identified potential risk factors using univariable conditional logistic regression models for each predictor, stratified by gender. Further, risk factors were assessed for whether there was a significant interaction between the risk factor and gender. Variables that were significant either for women and/or men at the α = 0.1 significance level or interactions that were found to be statistically significant at the α = 0.1 significance level were subsequently considered for the multivariable conditional logistic regression model developed using stepwise backwards elimination, stopping at explanatory variables that showed significance at α = 0.05. Prior to model development, we tested for collinearity between the variables of interest using Pearson correlation (r < 0.5). We did not find any variables collinear based on this test.

Because cases and controls were matched on age, sex and neighborhood, these variables were not included in the model. We analyzed the data using Stata v11.2 (StataCorp, College Station, TX, USA).

### Ethics

Informed verbal consent was sought and obtained from study respondents before their participation in the study. The name and contact of the respondent or study subjects were not included in our records. The study protocol was reviewed and approved by the School of Public Health’s institutional review board (IRB), “operating under the Rwanda National Ethics Committee.”

## Results

Of the 156 cases of homicide victims that were investigated between May 2011 and 2013, 89 (57 %) were men and 67(43 %) were women (Table 1). The mean age of homicide victims was 38.2 years (standard deviation (SD) = 14.9 years). The mean age by sex status was 36.6 (SD = 13.2 years) and 40.2 years (SD = 16.6 years) for male and female homicide victims, respectively. Most cases (133, 85.3 %) were residents of rural areas. More than half (81, 51.9 %) of homicides occurred inside the victim’s home, 53 (34.0 %) in the neighborhood (village of residence) and 22 (14.1 %) in a removed area. One hundred and forty victims (89.7 %) knew the alleged perpetrators, 53 (34.0 %) were acquaintances, 43 (27.6 %) were intimate partners that included former or current spouses, boyfriends or girlfriends of the victim. Of these intimate partner homicide victims, 31 (72.1 %) were women and 12 (27.9 %) were men. Further, there were 42 (26.9 %) homicide victims who were killed by family members, other than spouses. The great majority of victims (145, 92.9 %) underwent a postmortem examination. The most common reported mechanism of fatal injury was wounds inflicted by a knife or any other sharp instrument (64, 41.0 %), followed by blunt force (57, 36.5 %), and strangulation (17, 10.9 %).

**Table 1:** Description of characteristics of 156 homicide victims by gender status

Variables	Women	%	Men	%	Total	%
	(*n* = 67)		(*n* = 89)		(*N* = 156)	
Age group						
18–24	13	19.4	13	14.6	26	16.6
25–40	26	38.8	49	55.1	75	48.1
41–60	18	26.9	21	23.6	39	25
61–75	10	14.9	6	6.7	16	10.3
Province of residence						
Kigali	12	17.9	8	9	20	12.8
Southern	20	29.8	28	31.5	48	30.8
Eastern	12	17.9	24	26.9	36	23.1
Northern	5	7.5	7	7.9	12	7.7
Western	18	26.9	22	24.7	40	25.6
Area of residence						
Town/cities	8	11.9	15	16.8	23	14.7
Rural	59	88.1	74	83.2	133	85.3
Site of homicide						
Victim’s home	48	71.6	33	37.1	81	51.9
Neighborhood	15	22.4	38	42.7	53	34
Removed area	4	6	18	20.2	22	14.1
Time of homicide						
Morning/day hours	21	31.3	20	22.5	41	26.3
Evening	24	35.8	30	33.7	54	34.6
Night	22	32.8	37	41.6	59	37.8
Unknown	-	-	2	2.2	2	1.3
Relation of perpetrator to victim						
Acquaintance	9	13.4	44	49.5	53	34
Intimate partner	31	46.3	12	13.5	43	27.6
Family member	20	29.8	22	24.7	42	26.9
Stranger	1	1.5	1	1.1	2	1.2
Unidentified	6	9	10	11.2	16	10.2
Postmortem examination						
Was performed	60	89.5	85	95.5	145	93
Was not performed	5	10.5	6	4.5	11	7.1
Method of homicide						
Knife or sharp instrument	32	47.7	32	36	64	41
Blunt force	17	25.4	40	45	57	36.5
Strangulation	11	16.4	6	6.7	17	10.9
Poison	1	1.5	-		1	0.6
Handgun	1	1.5	2	2.2	3	1.9
Other	5	7.5	9	10.1	14	9

Table 2 shows the distribution of socio-demographic, criminological and genocide characteristics victims and controls by gender status. In the gender-stratified univariable logistic regression analysis, factors significantly associated with an increased risk of being a homicide victim in either women or men (at α = 0.10) included for both sex: polygamous marriage, having no surviving parents, victim slightly drinking and heavily drinking patterns and previous physical and/or sexual violence. For women only, the following factors were significant: dealing in illegal activities, such as prostitution, illegal selling of illicit brew and/or drug, history of past criminal record(s) and having first degree family member(s) who had been convicted of genocide. For men only, the following were significant: moderately drinking behavior and victim drinking intoxicating brew and/or drug use. On the contrary, the characteristic of belonging to a religion defined as being a member of a Christian church or being a member of Muslim congregation was significantly associated with lower crude odds of a death resulting from homicide in both women and men while having attained primary or secondary/tertiary education variables were significantly associated with unadjusted lower risk of homicide victimhood among men only.

**Table 2:** Univariate conditional logistic regression analysis of hypothesized risk factors by sex status of 156 cases of homicide victims and 468 living controls

	Women						Men						
	Cases		Control				Cases		Controls				
Variables	N	%	N	%	OR	*p*-value	N	%	N	%	OR	*p*-value	*p*-value for interaction
Marital status													
Single	10	14.9	30	14.9	1		29	32.6	87	32.6	1		
Married	31	46.3	125	62.2	0.8	0.778	42	47.2	148	55.4	0.9	0.860	0.888
Divorced/separated/widowed	12	17.9	35	17.4	1.0	0.995	3	3.4	4	1.5	4.1	0.174	0.267
Polygamous	14	20.9	9	4.5	6.7	0.022	15	16.8	23	8.6	2.6	0.080	0.345
Belonging to a religion													
No	14	20.9	2	1	1		17	19.1	22	8.2	1		
Yes	53	79.1	198	98.5	0.05	<0.001	72	80.9	242	90.6	0.4	0.006	0.018
Having children													
No	7	10.5	30	14.9	1		29	32.6	88	32.9	1		
Yes	60	89.5	171	85.1	1.9	0.238	60	67.4	178	66.7	1.1	0.902	0.352
Number of parents surviving													
Both parents	17	25.4	55	27.4	1		27	30.3	90	33.7	1		
One parent	22	32.8	83	41.3	0.8	0.697	27	30.3	112	42	0.9	0.638	0.978
No parent	28	41.8	63	31.4	2.2	0.079	35	39.4	63	23.6	2.7	0.010	0.781
Education level													
None	27	40.3	63	31.3	1		21	23.6	36	13.5	1		
Primary	36	53.7	121	60.2	0.6	0.159	64	71.9	202	75.7	0.4	0.020	0.518
Secondary or tertiary	3	4.5	16	8	0.3	0.143	3	3.4	29	10.8	0.1	0.003	0.416
Employment status													
Employed	56	83.6	176	87.6	1		78	87.6	237	88.7	1		
Dealing in illegal activities	7	10.4	5	2.5	4.9	0.025	2	2.3	9	3.4	0.6	0.563	0.076
Unemployed/other	3	4.5	20	9.9	0.4	0.244	9	10.1	17	6.4	1.9	0.208	0.099
Alcohol drinking patterns													
Not drinking	22	32.8	118	57.8	1		12	13.5	99	37.1	1		
Slightly	29	43.3	64	31.8	2.8	0.01	28	31.5	78	29.2	3.2	0.006	0.790
Moderately	4	6	15	22.4	2.1	0.276	29	32.6	54	20.2	5.2	<0.001	0.275
Heavily	12	17.9	4	6	39.9	0.001	20	22.4	36	13.5	6.1	<0.001	0.105
Previous gender based violence													
No	28	41.8	156	77.6	1		63	70.8	228	85.4	1		
Yes	35	52.2	32	15.9	16.5	<0.001	22	24.7	30	11.2	6.7	<0.001	0.214
Past criminal record(s)													
No	63	94	196	97.5	1		74	83.1	242	90.6	1		
Yes	4	6	3	1.5	4	0.070	13	14.6	25	9.4	1.8	0.135	0.370
Drinking intoxicating brew and/or drug use													
No	60	89.5	176	87.6	1		66	74.1	241	90.3	1		
Yes	4	6	2	0.1	5.6	0.146	21	23.6	16	6	5.8	<0.001	0.972
Genocide lasting effects													
Being a genocide survivor													
No	63	94	182	90.5	1		77	85.5	230	86.1	1		
Yes	4	6	19	9.5	0.6	0.382	12	13.5	37	13.9	0.9	0.922	0.507
Having lost first degree family member(s) to genocide													
No	64	95.5	180	89.5	1		80	89.9	231	86.5	1		
Yes	3	4.5	21	10.5	0.4	0.146	9	10.1	36	13.5	0.7	0.368	0.480
Having been convicted of genocide													
No	67	100	196	97.5	1		83	93.3	257	96.3	1		
Yes	0	0	5	2.5	-	-	6	6.7	10	3.7	2.4	0.179	-
Having first degree family member(s) who had been convicted of genocide													
No	46	68.7	156	77.6	1		74	83.1	226	84.5	1		
Yes	21	31.3	44	21.9	1.8	0.090	14	15.7	36	14.5	1.2	0.619	0.413
Having been a witness in a genocide trial													
No	61	91	175	87.1	1		78	87.6	223	83.5	1		
Yes	5	7.5	22	10.9	0.5	0.292	10	11.2	41	11.4	0.6	0.257	0.768

Prior to fitting our multivariable conditional logistic models, we further performed the test of interaction to identify factors that had statistically significant different effect on homicide victimhood according to gender status. Our results exhibited that belonging to a religion, dealing in illegal activities and being unemployed were the only factors that had a different effect on homicide victimization according to gender status.

In the final model, there were several factors with an adjusted higher risk of homicide victimization, regardless of the gender status (Table 3). Indeed, having no surviving parent significantly increased the odds for homicide victimization (aOR = 2.683, 95%CI: 1.083–6.650). The odds of homicide victimization for alcohol drinkers significantly increased with the frequency of drinking compared to non-drinkers: the adjusted odds ratio of study subjects increasing from those who drank beer slightly (roughly 1 day per week), aOR = 3.194 (95%CI: 1.296–7.871); moderately (2 to 3 days per week), aOR = 10.141 (95%CI: 3.744–27.463); and heavily (4 days and more per week), aOR = 11.542 (95%CI: 3.620–36.795). Previous physical, psychological and/or sexual abuse also remained strongly associated with victimization (aOR = 28.246, 95%CI: 9.557–83.476). Drug use and consumption of illicit intoxicating traditional brew also remained significantly associated with a higher risk of homicide victimhood compared to non–users (aOR = 7.671, 95%CI: 2.363–24.894). In contrast, belonging to a religion significantly reduced the odds of homicide victimization; the effect was significantly different for women and men, with aOR = 0.002 (95%CI: 0.001–0.054) and aOR = 0.200 (95%CI: 0.052–0.509), respectively.

**Table 3:** Multivariate conditional logistic regression analysis of hypothesized risk factors by sex status of 156 cases of homicide victims and 468 living controls

Variables	Adjusted OR	95%CI	*P*-value
Belonging to a religion			
Women			
No	1		
Yes	0.002	0.001–0.054	<0.001
Men			
No	1		
Yes	0.2	0.052–0.509	0.002
Number of parents surviving			
Both parents	1		
One parent	0.788	0.419–1.481	0.460
No parent	2.683	1.083–6.650	0.033
Alcohol drinking behavior			
Don’t drink	1		
Slightly	3.194	1.296–7.871	0.012
Moderately	10.141	3.744–27.463	<0.001
Heavily	11.542	3.620–36.795	<0.001
Previous gender based violence			
No	1		
Yes	28.246	9.557–83.476	<0.001
Drinking intoxicating brew and/or drug use			
No	1		
Yes	7.671	2.363–24.894	0.001

## Discussion

In this study, we found that the majority of homicide victims were male (57 %); however, the proportion of female victims (43 %) is considerably higher than what was observed in other studies and reports [1, 4, 16–22]. The most common cause of death was stab wounds, inflicted by knives or any other sharp instruments, followed by blunt force injuries. This result differed slightly from findings of a recent studies in South Africa that identified gunshot wounds as the most prevalent cause of homicide, followed by blunt force fatal injuries [23, 24].

Further, descriptive analysis showed that 43 (27.6 %) and 42 (26.9 %) of cases were killed by an intimate partner and a first-degree family member, respectively. Of the intimate partner homicides, 31 were women and 12 men, accounting for 46.3 % of all female homicide victims and 13.5 % of all male homicide victims. This was considerably higher than the 38.6 % of female and 6.3 % of male homicide victims who were killed by intimate partner, identified in recent systematic review of intimate partner homicides from 66 countries [12]. The increased vulnerability of Rwandan women, particularly in their own households should be further investigated and interventions to address this increased vulnerability should be prioritized. In 2009, Rwanda initiated a pilot project of a one-stop center, known as Isange (“feel welcome”, “feel at home”) for victims of child, domestic, or sexual abuse, or gender-based violence. The facility provides a holistic package of services to victims, ranging from a friendly reception to forensic medical assessment and evidence collection, prevention of diseases, psychological counseling, and awareness campaigns. The services are integrated into a public hospital and are provided to victims at no cost. The project is now being scaled up to all district hospitals of the country, and could be a sound strategy to fight gender-based violence.

We found that alcohol drinking behavior, previous gender based violence, consumption of homemade illicit brew and/or use of drug such as cannabis were surprisingly risk factors for homicide victimization in Rwanda post genocide regardless of gender status of homicide victims. Our results were consistent with findings of other studies, conducted in Tanzania and Kenya, two neighboring East African countries with relatively similar socio-economic situations [5, 17]. However, these risk factors are not specific to sub-Saharan African countries, as evidenced by the findings on homicide risk factors in Sweden and United States [1, 2]. We were surprised that the effect of alcohol was the same among women and men. We thought that this finding among women could be explained by their consumption of home-brewed alcohol sold illegally, especially in rural areas. This result is consistent with findings of a study conducted in South Africa that demonstrated high level of blood alcohol concentration among the majority of women homicide victims at the time of their death in Western Cape [25].

Further, as found in a homicide study in Dar es Salam, Tanzania, [8] we found that belonging to any religion reduced the odds of being killed among both men and women in Rwanda. Religion may be a proxy for other community and social factors, and the role of religion in protection against homicide must be further studied. However, the protective effect was more marked among females. Indeed, in our investigation, the gender based analysis revealed that out of all identified risk factors, belonging to a religion was the only factor that showed a statistically significant effect according to gender status of homicide victims.

Notably, our sex specific findings differed from studies conducted in South Africa and Western countries that extensively investigated intimate partner violence and firearm availability and female homicide [23–33]. Our study did not specifically study these two homicide risk factors, but rather our aim was to evaluate common socio-demographic characteristics of homicide victims and long lasting effects of genocide. Unlike South Africa and Western countries, the availability and use of firearms are legally limited to national security organs and few private security companies in Rwanda. Intimate partner violence was not well documented till 2009 when the Government established Isange one stop center. Our findings did identify previous physical and/or sexual violence as a risk factor for homicide victimization and more interesting, the characteristic did not any significant difference on homicide victimhood based on gender status. This finding is inconsistent with the results of a studies carried out in South Africa and Portugal that found prior domestic violence as a high risk factor for homicide victimhood among women [26–33]. However, while interpreting our results, the lack of significant difference of prior gender based violence on homicide victimization by gender status should be considered with caution because our study was not initially designed to specifically investigate intimate partner violence. Further, we recommend future studies in Rwanda and the region to better understand how this and other risk factors for homicide differs between men and women.

Prior to this study, we hypothesized that in addition to common well-documented risk factors for homicide victimhood, some genocide-related characteristics could increase risk of homicide victimization as a result of a feeling of revenge or deep social division between survivors and perpetrators in the aftermath of genocide. However, based on these results, no genocide-related exposures were significantly linked to the risk of homicide victimization, which could reflect the effectiveness of the Rwandan government’s policies to protect different pockets of Rwandan society, including survivors and witnesses from the 1994 Genocide. We believe that two policies, amongst many others, have been particularly instrumental in providing security to vulnerable groups. In the aftermath of the genocide, the government adopted a policy of rural human settlement in villages, known as “umudugudu,” defined as “a planned settlement made of between 100 and 200 houses by site in rural areas.” Thus, villages of vulnerable genocide survivors were constructed with government financial assistance, and their security was better provided [34]. Additionally, Rwanda National Police introduced community policing, a proactive community-based strategy whereby elected representatives in villages work with police liaison officers in identifying security issues, providing timely information that immediately dictates preventive measures [11].

### Limitations

There are several possible limitations to this study. First, it is likely that a small sample size could have reduced our ability to detect gender difference in the stratified analysis. The selection bias may have been introduced into this study. Indeed, homicide victim definition was based on the police report, not on forensic medical autopsies that are only performed in the capital city of Kigali. Additionally, we limited our investigation to homicide victims whose families were traced, consented to participate in the study and willingly agreed to provide information on the deceased. The selection of controls was not randomly performed, but rather we relied on matching criteria and the assistance of the village administrators. In order to minimize the effect of non–randomization of living controls, interviewers sensitized the village administrators to reduce the preferential selection of certain individuals.

There is also potential for information bias resulting from the information being obtained from a third party (family respondent). Respondents may have different levels of familiarity with study subjects, which may have affected their ability to equally remember their characteristics. Respondents could also have willingly provided deceptive information for fear of being associated with the ongoing criminal investigation by the police. Data collectors tried to validate responses from third sources when information was inconsistent and if the primary informant seemed unreliable.

## Conclusion

In Rwanda, homicide victims are relatively young and the proportion of female victims is one of the highest in the world. Even in the context of residual from a mass genocide, homicide victimization risk factors are not unique. Sensitizing communities against heavy alcohol drinking and drinking homemade illicit brew and/or drug use and fighting gender based violence could reduce the rate of homicide deaths among Rwandans. Future research should explore the protective mechanism of religion against homicide death in Rwanda post genocide. These findings are valuable for the development of strategies to prevent homicide death in post-genocide Rwanda.

In Rwanda, because medico-legal standardized reporting or active fatal injury surveillance systems do not exist, the collaboration between public health researchers and law enforcement organizations offered an appropriate framework that is conducive to research on this highly sensitive subject [13, 35–37]. This new and emerging field of forensic epidemiology, which lies at the intersection of public health and law, could play an important role in studying and understanding characteristics and risk factors of health-related criminal events in developing countries. This would be an important step in developing preventive strategies and effective measures for reducing homicide.

## References

[1] Allgulander C, Nilsson B (2000). Victims of criminal homicide in Sweden: a matched case- control of health and social risk factors among all 1,739 cases during 1978–1994. Am J Psych.

[2] Weibe DJ (2003). Homicide and suicide risks associated with firearms in the home: a national case–control study. Ann of Emerg Med.

[3] Mian A, Mahmood SF, Chotani H, Luby S (2002). Vulnerability to homicide in Karachi. Int J Epidemiol.

[4] Dahlberg L, Ikeda RM, Kresnow M (2004). Guns in the home and a risk of violent death: findings from a national study. Am J Epidemiol.

[5] Outwater AH, Cambell JC, Mgaya M, Abraham AG, Kinabo L, Kazaura M (2005). Homicide deaths in Dar es Salaam, Tanzania. Int Journ Inj Contr Saf Promot.

[6] Mohanty S, Sen M, Sahu G (2013). Analysis of risk factors of dowry-death- a South Indian study. J Forensic Leg Med.

[7] Silva MA, Cabral Filho JE, Amorim MM, Falbo Neto GH (2013). Female homicide victims in Recife, Pernambuco State, Brazil, 2009–2010: a descriptive study. Cad Saude Publica.

[8] Kibusi SM, Ohnishi M, Outwater A, Seino K, Kizuki M, Takano T (2013). Sociocultural factors that reduce risk of homicide in Dar es Salaam: a case control study. Inj Prev.

[9] Hansen T (2005). The Gacaca tribunals in post-genocide Rwanda. Center for restorative justice & peacemaking.

[10] Rieder H, Elbert T (2013). Rwanda – lasting imprints of a genocide: trauma, mental health and psychosocial conditions in survivors, former prisoners and their children. Confl Health.

[11] Rwanda National Police (2014). Policing a rapidly transforming post-genocide society: Making Rwandans feel safe, involved and reassured.

[12] Stöckl HS, Devries K,Rotstein A,Abrahams A,Campbell J et al. The global prevalence of intimate partner homicide: a systematic review. The Lancet 2013.Available online at: http://www.thelancet.com/pdfs/journals/lancet/PIIS0140-6736(13)61030-2.pdf, date last accessed 10 June 2015 10.1016/S0140-6736(13)61030-2 23791474

[13] Organic law instituting the penal code (2012). No 01/2012/OL of 02/05/2012.

[14] United Nations Security Council. Security Council Adopts Resolution 2136, Renewing Arms Embargo, Related Measures Imposed on Democratic Republic of Congo. New York: Press Statement; 2014. Available online at: http://www.un.org/press/en/2014/sc11268.doc.htm, date last accessed 18 August 2015.

[15] Sosnov M, Ball H (2008). The adjudication of genocide: Gacaca and the road to reconciliation in Rwanda. J Intern Law and Policy.

[16] Cardona M, Garcia HI, Giraldo CA, López MV, Suárez CM, Corcho DC (2005). Homicides in Medellin, from 1990 to 2002: victims, motives and circumstances. Cad Saude Publica.

[17] Ziraba AK, Kyobutung C, Zulu EM (2011). Fatal injuries in the slums of Nairobi and the risk factors: results from a matched case–control study. J Urban Health.

[18] Meel BL (2008). Homicides trends in the Mthatha area between 1993 and 2005. S Afr Med J.

[19] Cooper A, Smith EL (2011). Homicide trends in the United States, 1980–2008.

[20] Office for National Statistics (2013). Statistical bulletin.

[21] United Nations office on drug and crimes (UNODC) (2014). Some 437,000 people murdered worldwide in 2012,according to new UNODC study.

[22] Lim M, Ganpat S, Granath S, Hangstedt J, Kivivuori J, Lehti M (2013). Homicide in Finland, the Netherlands and Sweden: First findings from the European monitor. Homicide Stud.

[23] Cocks J, Saayman G (2013). The incidence, pathology of trauma and victim profiles of homicide death in Pretoria, South Africa (2007–2008). Med Sci Law.

[24] Matzopulos RG, Thompson ML, Myers JE (2014). Firearm and non-firearm in South African cities: a retrospective population-based study. Am J Public Health.

[25] Mathews S, Abrahams N, Jewkes R, Martin LJ, Lombard C (2009). Alcohol use and its role in female homicides in the Western Cape, South Africa. J Stud Alcohol Drugs.

[26] Abrahams N, Jewkes R, Mathews S (2010). Guns and gender based violence in South Africa. S Afr Med J.

[27] Mathews S, Abrahams N, Jewkes R, Martin LJ, Lombard C, Vetten L (2009). Injury patterns of female homicide victims in South Africa. J Trauma.

[28] Abrahams N, Mathews S, Jewkes R, Martin L, Vetten L (2006). The role of guns in domestic violence: evidence from South Africa.

[29] Lamb G (2008). ‘Under the gun’: An assessment of firearm crime and violence in South Africa.

[30] Chetty R (2002). Firearm use and distribution in South Africa.

[31] Abrahams N, Jewkes R, Martin LJ, Mathews S, Vetten L, Lombard C (2009). Mortality of women from intimate partner violence in South Africa: a national epidemiological study. Violence Vict.

[32] Suffla S, Van Niekerk A, Arendse N (2008). Female homicidal strangulation in urban South Africa. BMC Public Health.

[33] Pereira AR, Viera DN, Magalhaes T (2013). Fatal intimate partner women in Portugal: a forensic medical national study. J Forensic Leg Med.

[34] Ministry of Infrastructure (2009). Updated version of the national human settlement in Rwanda.

[35] Goodman RA, Munson JW, Dammers K, Lazzarani Z, Barkley JP (2003). Forensic epidemiology: Law at the intersection of public health and criminal investigations. J Law Med Ethics.

[36] Loue S (1999). Forensic Epidemiology: A Comprehensive Guide for Legal and Epidemiology Professionals.

[37] Jernigan DB, Raghunathan PL, Bell BP, Brechner R, Bresnitz EA, Jay C (2001). Investigation of bioterrorism-related Anthrax, United States, 2001: Epidemiologic findings. Emerg Infect Dis.

